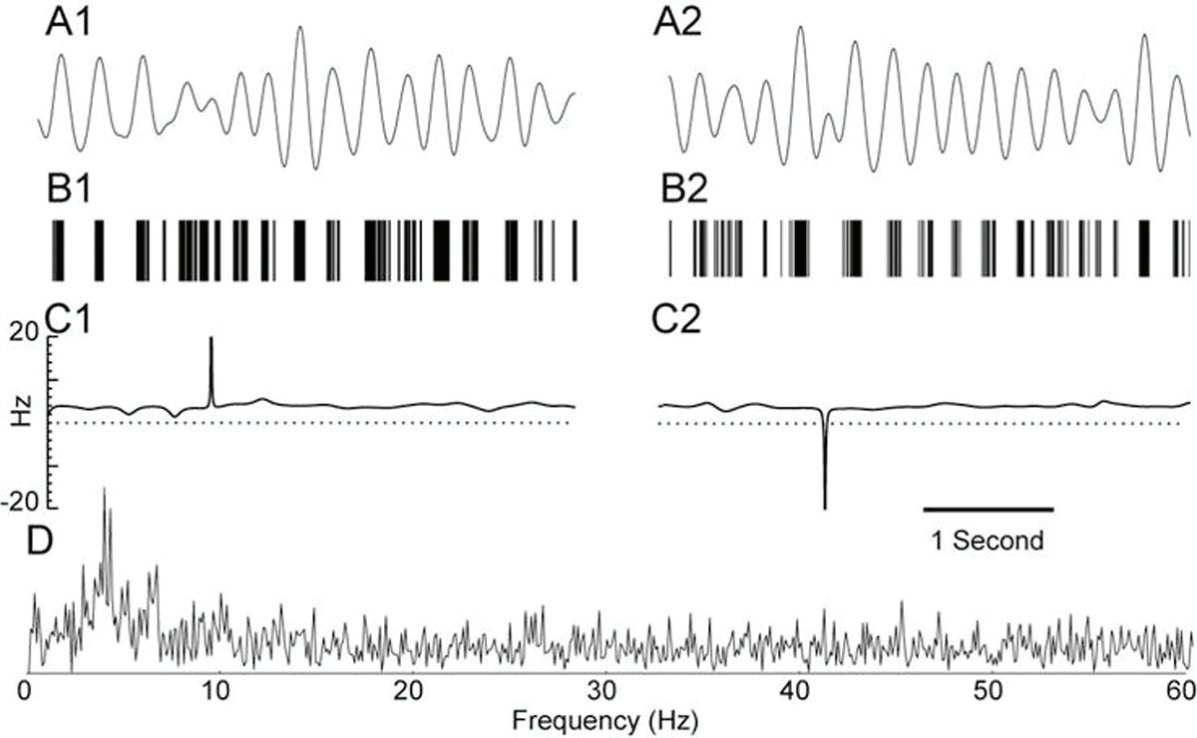# Phase locking and resetting in human subthalamic neurons

**DOI:** 10.1186/1471-2202-12-S1-P28

**Published:** 2011-07-18

**Authors:** Shuoguo Wang, Ted Weyand, Erich Richter, Carmen C Canavier

**Affiliations:** 1 Neuroscience Center of Excellence, LSUHSC, New Orleans, LA 70112, USA

## 

Microelectrode recordings were obtained from human subthalamic nucleus (STN) in parkinsonian patients using a “Ben gun” array of 5 microelectrodes [1] prior to implantation of a deep brain stimulation electrode, and digitized at 25 kHz. Neuronal spikes were threshold extracted (>4SD above baseline). Single units were confirmed using a spike sorting algorithm [2]. Spike trains were then constructed at 1 kHz with unit impulses at each spike time (Fig. 1B) and zeros elsewhere. Episodes of tremor related activity were identified in which the spectral peak in the tremor range (2-6 Hz) was substantially higher than baseline (Fig. 1D). Spike trains corresponding to identified oscillatory activity were then band pass filtered (2 to 6 Hz) to produce a continuous signal (Fig. 1A) corresponding to the envelope of the low frequency activity [3]. The Hilbert transform was used to reconstruct the phase of this signal at each time point. The derivative of this waveform gives the instantaneous frequency (Fig. 1C), and large deviations from the baseline rate of about 4 Hz indicate that the phase of the oscillation has been reset. We identified spontaneous phase advances (Fig. 1A1,B1, and C1) in which the next burst occurred sooner than expected as well as spontaneous phase delays (Fig. 1A2,B2, and C2)in which the next burst occurred later than expected. Our goal is to better understand abnormal synchronization in the STN.

**Figure 1:**